# Panic disorder and locomotor activity

**DOI:** 10.1186/1751-0759-2-23

**Published:** 2008-11-18

**Authors:** Noriyuki Sakamoto, Kazuhiro Yoshiuchi, Hiroe Kikuchi, Yoshiyuki Takimoto, Hisanobu Kaiya, Hiroaki Kumano, Yoshiharu Yamamoto, Akira Akabayashi

**Affiliations:** 1 Department of Stress Sciences and Psychosomatic Medicine, Graduate School of Medicine, the University of Tokyo, Tokyo, Japan; 2 Panic Disorder Research Center, Warakukai Medical Corporation, Tokyo, Japan; 3 Educational Physiology Laboratory, Graduate School of Education, the University of Tokyo, Tokyo, Japan

## Abstract

**Background:**

Panic disorder is one of the anxiety disorders, and anxiety is associated with some locomotor activity changes such as "restlessness". However, there have been few studies on locomotor activity in panic disorder using actigraphy, although many studies on other psychiatric disorders have been reported using actigraphy. Therefore, the aim of the present study was to investigate the relationship between panic disorder and locomotor activity pattern using a wrist-worn activity monitor. In addition, an ecological momentary assessment technique was used to record panic attacks in natural settings.

**Methods:**

Sixteen patients with panic disorder were asked to wear a watch-type computer as an electronic diary for recording panic attacks for two weeks. In addition, locomotor activity was measured and recorded continuously in an accelerometer equipped in the watch-type computer. Locomotor activity data were analyzed using double cosinor analysis to calculate mesor and the amplitude and acrophase of each of the circadian rhythm and 12-hour harmonic component. Correlations between panic disorder symptoms and locomotor activity were investigated.

**Results:**

There were significant positive correlations between the frequency of panic attacks and mesor calculated from double cosinor analysis of locomotor activity (r = 0.55) and between HAM-A scores and mesor calculated from double cosinor analysis of locomotor activity (r = 0.62).

**Conclusion:**

Panic disorder patients with more panic attacks and more anxiety have greater objectively assessed locomotor activity, which may reflect the "restlessness" of anxiety disorders.

## Background

Panic disorder is one of the anxiety disorders. Anxiety is associated with locomotor activity as described in some assessment tools for anxiety, such as the Hospital Anxiety and Depression Scale [1] and the Hamilton Anxiety Scale (HAM-A) [2], which includes items related to "restlessness".

There has been only one group investigating locomotor activity that objectively assessed patients with panic disorder. Clark et al. [3] reported that mean daily activity was higher in patients without phobic avoidance than in controls. The device for recording locomotor activity used in this previous study was attached to the lateral thigh, measured 4 × 8 × 12 cm and weighed 0.5 kg, and recorded physical activity categorized to eight levels. Therefore, the device would not be able to detect subtle changes in locomotor activity such as restlessness.

In other psychiatric diseases, such as seasonal affective disorder, major depressive disorder and attention-deficit hyperactivity disorder, wrist-worn activity monitors have been used to investigate the locomotor activity pattern and have successfully detected changes in the locomotor activity pattern in these psychiatric diseases [4-6].

Therefore, the aim of the present study was to investigate for two weeks the relationship between panic disorder and locomotor activity pattern using a wrist-worn activity monitor. In addition, in order to avoid recall bias panic attacks were recorded in a watch-type computer momentarily instead of interviewing patients. Recording data such as symptoms and physical activity momentarily in natural settings have been developed as an ecological momentary assessment (EMA) technique [7-9]. We hypothesized that patients with more severe symptoms such as more frequent attacks and more anxiety had greater amplitude as well as mesor and that there may be some relationship between more severe symptoms and the acrophase of locomotor activity because there were some studies on change in the circadian rhythm of physiological markers or symptoms in panic disorder [10-12].

## Methods

All the procedures and materials were approved by the institutional review board of the University of Tokyo and of the Warakukai Incorporated Medical Institution, and written informed consent was obtained from all subjects before the study.

### Subjects

Sixteen patients with panic attacks were recruited for the study through advertisements placed on the website and in the medical office of the Research **C**enter of Panic Disorder (Akasaka Clinic) and on the website of our department. Patients who applied for participation were interviewed and screened by a well-trained physician (N.S.).

Inclusion criteria were as follows: diagnosis of panic disorder according to DSM-IV-TR criteria [13]; at least one panic attack during each of the last two weeks; and age ≥ 20 but ≤ 50 years. Exclusion criteria were: current other psychiatric disease assessed by the Mini-International Neuropsychiatric Interview for DSM-IV criteria [14]; personality disorder assessed by the Structured Clinical Interview for DSM-IV criteria [15]; and severe physical illness. Patients were allowed to take medications, but not allowed to change medications during the recording period.

### Measurements

#### Psychological variables

The overall severity of panic disorder was measured by the total score on the clinician-rated Panic Disorder Severity Scale (PDSS) [16,17]. In addition, HAM-A [2] was used for assessment of general clinical severity. The two scales were performed **on **the first day of the present study.

#### Frequency of panic attacks

To record momentary panic attack intensity, watch-type computers (Ruputer ECOLOG; 42 g, Seiko Instruments Inc., Tokyo, Japan) were used as electronic diaries [8,9]. The computer was equipped with a screen measuring 20 × 30 mm. and a joystick and button as input devices. Subjects were fully instructed how to use the device and given manuals before the beginning of the study period. They also practised manipulating the device with one of the authors (N.S.) until they became accustomed to its use.

Subjects wore the watch-type computers for 14 consecutive days. Event-contingent recordings were defined as recordings that were initiated by the subjects themselves when a particular event occurred [7]. In this study, subjects were asked to make a recording every time they had a panic attack as an event-contingent recording.

In the electronic diaries, subjects were asked questions about subjective physical symptoms. The questions were 18 items arranged to evaluate 13 panic symptoms according to DSM-IV-TR criteria. The eighteen panic symptom items included "palpitation", "sweating", "shaking", "feeling of choking", "sensations of smothering", "chest pain", "abdominal distress", "feeling dizzy", "feeling unsteady", "feeling faint", "feelings of unreality", "depersonalization", "fear of going crazy", "fear of dying", "numbness", "tingling sensations", "chills", and "hot flushes". Subjective symptoms were rated on 21-point visual analogue scales (VAS) from 0 to 100 displayed on the screen. Each word for a subjective symptom was displayed with the VAS as a question, and the anchor words 'none' and 'most intense' were displayed at the respective ends of the scale. By manipulating a joystick, the subjects adjusted the length of the bar so that it corresponded to their subjective symptom intensity at that moment. Panic attacks were verified by confirming that the symptoms recorded satisfied the criteria of panic attacks according to DSN-IV-TR criteria.

Because the recording periods were different among patients due to battery problems or patient convenience, the frequency of panic attacks was calculated by dividing panic attacks by the recording period instead of simply using the number of panic attacks.

#### Physical activity

Wrist activity monitors were built into the watch-type computers used for recording panic attacks and worked synchronously with the computers. They were worn all day and night on the non-dominant hand for 14 consecutive days. The instrument was removed for bathing, showering or any other activity likely to cause water damage. The time the instrument was taken off and put back on was recorded by selecting 'taking off' or 'putting on' from the menu. The activity monitors are uni-axial piezo-electronic accelerometers with a sensitivity of 0.01 g, and are analogous in performance to the Actigraph Mini-Motionlogger (Ambulatory Monitors Inc., Ardsley, NY, USA), which has frequently been used in studies of physical activity. Zero-crossing mode was used and acceleration counts were accumulated for every epoch of 1 min.

#### Data analysis

Cosinor analysis was used to assess the timing and amplitude of **the **circadian rhythm of physical activity. Because the duration of the daytime activity period is greater than the nighttime quiescent period, traditional cosinor analysis is not suitable for analyzing physical activity data. Therefore, double cosinor analysis was used in the present study, in which the simultaneous fit of two cosine functions was performed, one with a period of 24 hours and the other with a 12-hour period [6,18]. This model yields estimates of mesor (corrected mean) and the amplitude and acrophase (time of the peak of the fit rhythm) of the circadian rhythm and 12-hour harmonic component. According to previous studies [6,18], activity counts at 5-min intervals were analyzed using double cosinor analysis.

#### Statistical analysis

Pearson's correlation analysis was used to investigate the relationship between panic disorder symptoms (frequency of panic attacks, PDSS, HAM-A) and locomotor activity variables calculated from the double cosinor analysis.

## Results

### Patient characteristics (Table 1)

**Table 1:** Demographic and medical characteristics of the subjects

sample size	n = 16 (male: n = 2, female: n = 14)
Age* (years)	32.8 ± 5.2
Diagnosis	PD with Agoraphobia: n = 16, PD without Agoraphobia: n = 0
Duration of panic disorder^† ^(months)	61 (1 – 267)
PDSS*	12.0 ± 3.6
HAM-A*	21.1 ± 8.4
Recording periods^† ^(days)	14 (11–18)
Number of recorded panic attacks^†^	3.5 (1–10)

* Variables are shown as mean ± SD.

^† ^Variables are shown as median (minimum – maximum).

PD, panic disorder; PDSS, Panic Disorder Severity Scale; HAM-A, the Hamilton Anxiety Scale.

All patients met criteria for panic disorder with agoraphobia according to DSM-IV criteria. The patients consisted of two male and 14 female patients (32.8 ± 5.2 years). All the patients completed their recordings for at least 11 days (range, 11 to 18 days, median 14 days). All the patients recorded at least one panic attack during their recordings (range, 1 to 10 attacks, median 3.5 attacks). Three patients took no medication, four patients took a benzodiazepine, eight patients took a selective serotonin reuptake inhibitor (SSRI) and a benzodiazepine, and one patient took a SSRI, a tricyclic antidepressant, and a benzodiazepine.

### Correlations between panic disorder symptoms and locomotor activity variables from dual cosinor analysis (Table 2)

**Table 2:** Correlations between panic disorder symptoms and locomotor activity (n = 16)

	PDSS		HAM-A		Frequency	
			
	r	p value	r	p value	r	p value
Mesor	0.71	0.79	0.62	0.01	0.55	0.03
Amplitude (circadian)	0.22	0.41	0.35	0.19	0.16	0.55
Amplitude (12 hour harmonic)	0.00	1.00	0.42	0.10	0.46	0.08
Acrophase (circadian)	0.13	0.62	0.22	0.42	0.36	0.18
Acrophase (12 hour harmonic)	-0.27	0.31	-0.18	0.51	-0.18	0.49

PDSS, Panic Disorder Severity Scale; HAM-A, the Hamilton Anxiety Scale; Frequency, number of panic attacks per day during the recording period.

**Figure 1:**
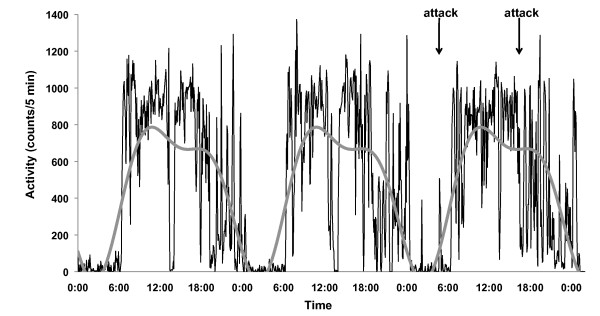
**Representative data for locomotor activity and a fitted line from double cosinor analysis**. The shaggy black line graph shows physical activity counts per 5 minutes. The grey smooth line graph shows a fitted curve calculated from double cosinor analysis. The arrows show the times when panic attacks occurred.

Representative data for locomotor activity and a fitted line from double cosinor analysis are shown in Figure 1.

**Figure 2:**
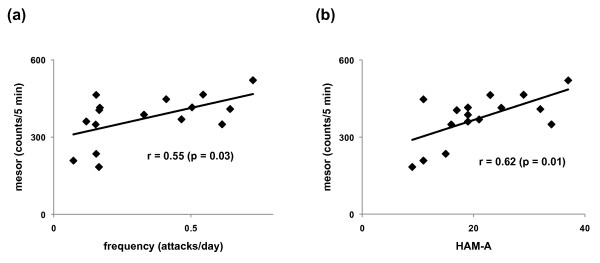
**Scatter plots of mesor and frequency of panic attacks and HAM-A scores**. (a) A scatter plot of mesor and frequency of panic attacks per day. There was a significant positive correlation (r = 0.55, p = 0.03). (b) A scatter plot of mesor and HAM-A scores. There was a significant positive correlation (r = 0.62, p = 0.01).

There were significant positive correlations between the frequency of panic attacks and mesor calculated from the double cosinor analysis of locomotor activity (r = 0.55, p = 0.03) and between HAM-A scores and mesor calculated from the double cosinor analysis of locomotor activity (r = 0.62, p = 0.01) (Figure 2). There were no significant correlations between any other variables and the frequency of panic attacks.

## Discussion

In the present study, significant positive correlations were found between the frequency of panic attacks and mesor calculated from the double cosinor analysis of locomotor activity and between Hamilton Anxiety Scale scores and mesor calculated from the double cosinor analysis of locomotor activity, which means that patients with more panic attacks and with more anxiety have greater mean locomotor activity levels.

The results of the present study are consistent with those of the previous study by Clark et al. [3], which showed that mean daily activity was higher in patients without phobic avoidance than in controls. However, the comorbidity of agoraphobia was different between the present study and the previous study. Therefore, it is difficult to compare the results between the two studies.

Previous studies showed that physical activity might be a trigger for panic attacks in patients with panic disorder [19,20], which implies the possibility that higher locomotor activity might cause more panic attacks in the present study. However, Broman-Fulks et al. [21] recently reported that aerobic exercise reduced anxiety sensitivity. Therefore, further studies on the relationship between physical activity and anxiety are needed.

There was no significant correlation between amplitude and attack frequency or HAM-A or between acrophase, and attack frequency or HAM-A in the present study, contrary to our hypothesis, which suggests that more severe symptoms are not related to greater diurnal variation of locomotor activity or with the acrophase of locomotor activity. Therefore, the results in the present study suggest that patients with more severe symptoms had greater locomotor activity evenly throughout day and night without increased diurnal variation and without change in acrophase.

There are some limitations in the present study. First, the sample size was relatively small. Second, all patients took medications such as SSRI. Therefore, it is difficult to exclude the influence of medications on the relationship between panic attacks and locomotor activity, although the medications that the patients took did not change during the recording period. Third, all our patients had agoraphobia. Therefore, it is not clear if these results can be applied to patients without agoraphobia. Fourth, the temporal relationship between panic attacks and locomotor activity could not be investigated by cosinor analysis. Therefore, further studies on temporal relationship between panic attacks and locomotor activity are necessary to investigate the influence of panic attacks on locomotor activity. Finally, there were no healthy controls in the present study. Therefore, further studies are needed to investigate the difference in locomotor activity between panic disorder patients and healthy controls.

## Conclusion

Panic disorder patients with more panic attacks and more anxiety have greater objectively assessed locomotor activity, which may reflect the symptoms of anxiety disorders.

## Competing interests

The authors declare that they have no competing interests.

## Authors' contributions

NS designed the study, collected the data, interpreted the results, and drafted the manuscript. KY designed the study, analyzed the data, performed the statistical analysis, interpreted the results, and drafted the manuscript. YT designed the study, interpreted the results and drafted the manuscript. HK1 analyzed the data, performed the statistical analysis, interpreted the results, and drafted the manuscript. YY helped analyze the data, interpret the results, and draft the manuscript. HK2, HK3, and AA helped interpret the results, and draft the manuscript. All authors read and approved the final manuscript.
